# Genomic surveillance of *Escherichia coli* and *Klebsiella* spp. in hospital sink drains and patients

**DOI:** 10.1099/mgen.0.000391

**Published:** 2020-06-18

**Authors:** Bede Constantinides, Kevin K. Chau, T. Phuong Quan, Gillian Rodger, Monique I. Andersson, Katie Jeffery, Sam Lipworth, Hyun S. Gweon, Andy Peniket, Graham Pike, Julian Millo, Mary Byukusenge, Matt Holdaway, Cat Gibbons, Amy J. Mathers, Derrick W. Crook, Timothy E.A. Peto, A. Sarah Walker, Nicole Stoesser

**Affiliations:** ^1^​ Nuffield Department of Medicine, University of Oxford, Oxford, UK; ^2^​ National Institute for Health Research (NIHR) Health Protection Research Unit on Healthcare Associated Infections and Antimicrobial Resistance, John Radcliffe Hospital, Oxford, UK; ^3^​ NIHR Biomedical Research Centre, Oxford, UK; ^4^​ Department of Infectious diseases/Microbiology, Oxford University Hospitals NHS Foundation Trust, Oxford, UK; ^5^​ Harborne Building, School of Biological Sciences, University of Reading, Reading, UK; ^6^​ Department of Haematology, Oxford University Hospitals NHS Foundation Trust, Oxford, UK; ^7^​ Department of Medicine, Oxford University Hospitals NHS Foundation Trust, Oxford, UK; ^8^​ Adult Intensive Care Unit, Oxford University Hospitals NHS Foundation Trust, Oxford, UK; ^9^​ Clinical Microbiology, Department of Pathology, University of Virginia Health System, Charlottesville, Virginia, USA; ^10^​ Division of Infectious Diseases and International Health, Department of Medicine, University of Virginia Health System, Charlottesville, Virginia, USA

**Keywords:** antibiotic resistance, Enterobacterales, resistome, sinks, wastewater

## Abstract

*
Escherichia coli* and *Klebsiella* spp. are important human pathogens that cause a wide spectrum of clinical disease. In healthcare settings, sinks and other wastewater sites have been shown to be reservoirs of antimicrobial-resistant *E. coli* and *Klebsiella* spp., particularly in the context of outbreaks of resistant strains amongst patients. Without focusing exclusively on resistance markers or a clinical outbreak, we demonstrate that many hospital sink drains are abundantly and persistently colonized with diverse populations of *E. coli *, *Klebsiella pneumoniae* and *Klebsiella oxytoca *, including both antimicrobial-resistant and susceptible strains. Using whole-genome sequencing of 439 isolates, we show that environmental bacterial populations are largely structured by ward and sink, with only a handful of lineages, such as *E. coli* ST635, being widely distributed, suggesting different prevailing ecologies, which may vary as a result of different inputs and selection pressures. Whole-genome sequencing of 46 contemporaneous patient isolates identified one (2 %; 95 % CI 0.05–11 %) *E. coli* urine infection-associated isolate with high similarity to a prior sink isolate, suggesting that sinks may contribute to up to 10 % of infections caused by these organisms in patients on the ward over the same timeframe. Using metagenomics from 20 sink-timepoints, we show that sinks also harbour many clinically relevant antimicrobial resistance genes including *bla*_CTX-M_, *bla*_SHV_ and *mcr*, and may act as niches for the exchange and amplification of these genes. Our study reinforces the potential role of sinks in contributing to Enterobacterales infection and antimicrobial resistance in hospital patients, something that could be amenable to intervention. This article contains data hosted by Microreact.

## Data Summary

Raw sequencing data are available under NCBI SRA accessions PRJNA604910 and PRJNA604975 (cultured isolates), and ENA project PRJEB36775 (metagenomes). A supporting data repository containing metadata, phylogenies, Jupyter notebooks and Pavian reports is archived at Figshare (doi:10.6084/m9.figshare.12337283). Microreact projects are available for E. coli, K. oxytoca and K. pneumoniae cultures. The authors confirm all supporting data, code and protocols have been provided within the article or the supporting data repository.

**Impact Statement**

*
Escherichia coli* and *Klebsiella* spp. cause a wide range of bacterial infections, including bloodstream, urine and lung infections. Previous studies have shown that sink drains in hospitals may be part of transmission chains in outbreaks of antimicrobial-resistant *E. coli* and *Klebsiella* spp., leading to colonization and clinical disease in patients. We show that even in non-outbreak settings, contamination of sink drains by these bacteria is common across hospital wards, and that many antimicrobial resistance genes can be found and potentially exchanged in these sink drain sites. Our findings demonstrate that the colonization of handwashing sink drains by these bacteria in hospitals is likely contributing to some infections in patients, and that additional work is needed to further quantify this risk, and to consider appropriate mitigating interventions.

## Introduction

Infections caused by Enterobacterales, including *Escherichia coli* and *Klebsiella* spp., are major causes of global morbidity, and particular antimicrobial-resistant strains (namely extended-spectrum beta-lactamase [ESBL] and carbapenemase producers) have been listed as critical priority pathogens for mitigation by the World Health Organization. In the UK, year-on-year increases have been observed in the number of *E. coli* and *Klebsiella* spp. bloodstream infections [1] for reasons that remain unclear. As well as causing invasive disease, these organisms are capable of colonizing a wide range of animal and environmental niches, and are frequently carried in the human gastrointestinal tract [2]. As such, they are also commonly found in human wastewater, and in wastewater-associated sites such as sewers and water-treatment infrastructure [3].

A significant proportion of Enterobacterales infections are healthcare-associated, prompting the UK government to introduce a target in 2016 to halve the number of healthcare-associated Gram-negative bloodstream infections by 2021 [4]. Wastewater sites in hospitals have been highlighted as reservoirs of drug-resistant Enterobacterales, with several studies reporting that ongoing transmission and outbreaks of human disease are associated with the contamination of, for example, sinks, by these organisms [5, 6]. More recently, several studies have shown reductions in colonization and/or invasive infection with Enterobacterales and other Gram-negative bacilli following the introduction of strategies to remove sinks and mitigate possible contamination from wastewater sources in patient rooms [7, 8]. Most of these studies however focus on the sampling and control of antimicrobial-resistant strains, often representing a more immediate clinical problem in an outbreak setting, rather than on the possibility that these sites may represent part of the wider endemic transmission network of both susceptible and resistant strains causing infection in patients.

Whole-genome sequencing of bacterial isolates is increasingly used as the most robust, high-resolution approach to characterizing relatedness between strains, and hence determining likely transmission [9]. However, the diversity of complex, polymicrobial environmental reservoirs is incompletely captured by sequencing small numbers of isolates, and this breadth of diversity can be more fully captured by using a metagenomic approach, which characterizes the genetic complement of a whole sample [10]. Combining both approaches has been shown to improve our understanding of species and antimicrobial resistance (AMR) gene diversity within environmental, wastewater and river samples [11, 12] and of transmission in a sink-associated outbreak of *Sphingomonas koreensis* (also a Gram-negative bacillus) in the NIH Clinical Centre in the USA [13].

In order to investigate the prevalence of contamination of healthcare sinks by strains of *E. coli* and *Klebsiella* spp., including those resistant to third-generation cephalosporins and carbapenems, we sampled all sink sites using p-trap (U-bend) aspirates across several wards and timepoints in a single UK hospital in 2017. We used a combination of whole-genome sequencing of cultured isolates from all sink samples and metagenomic sequencing of a subset of sink samples to facilitate a high-resolution assessment of the genetic diversity present in these niches. To determine whether sinks were a reservoir of Enterobacterales strains causing infection in patients over similar timeframes, we simultaneously retrieved relevant isolates from culture-positive specimens taken from patients admitted to the same ward locations, and used genomics to identify the degree of genetic relatedness.

## Methods

### Ward-based sink sampling

We sampled three units (acute admissions [AA], adult critical care [ACC], adult general medicine [female only] [GM]) within a single hospital (John Radcliffe Hospital, Oxford, UK) four times on rotation every 3 weeks over 3 months, March–May 2017. Units were chosen to capture different patient populations, admission turnaround times and wastewater plumbing infrastructure. The haematology ward (on a separate hospital site) was also sampled on a single day (12 May 2017) subsequent to a small cluster of patient cases of *bla*_OXA-48_ carbapenemase-associated Enterobacterales bloodstream infections described previously [14]. Ward and sink/wastewater layouts were obtained from estates, and each sink/drain site was assigned a unique site identifier (Table S1).

On each day of sampling, autoclaved tubing cut to 10 inches was used to aspirate from sink p-traps via a sterile 50 ml syringe. Up to 50 ml of fluid was aspirated where possible. Then, 100 µl of tenfold dilutions (10^−2^, 10^−3^, 10^−4^) of each sink p-trap aspirate were plated onto CHROMagar Orientation media (Becton Dickinson, Franklin Lakes, NJ, USA), with no disc, cefpodoxime (10 µg), ertapenem (10 µg) (Thermo Scientific Oxoid, Basingstoke, UK) applied in a triangular fashion to each plate. Cultures were incubated at 37 °C for ~18 h. Growth of Enterobacterales (presence/absence) and density (sparse/dense/confluent) in all zones was recorded (i.e. no antibiotic, in the presence of cefpodoxime, and in the presence of ertapenem). Up to four distinct colonies of each of presumptive *E. coli* and *Klebsiella* spp. were sub-cultured on CHROMagar Orientation to confirm purity and species identification. Species identification of sub-cultured colonies was confirmed by MALDI-ToF (MALDI Biotyper, Bruker, Billerica, MA, USA). Stocks of sub-cultured isolates were stored at −80 °C in 400 µl of nutrient broth +10 % glycerol prior to DNA extraction for sequencing. Aspirates from sink p-traps were then centrifuged at 4000 r.p.m. for 10 min at 4 °C, and supernatants removed; pellets were stored at −80 °C.

### Patient isolate sampling

For AA, ACC and GM wards, a pseudo-anonymized, prospective feed was set-up to try and enable real-time capture of isolates from all samples culture-positive for *E. coli *, *K. pneumoniae* and *K. oxytoca* from patients admitted to any of these wards during the study time period, and were processed routinely through the clinical microbiology laboratory in the John Radcliffe Hospital in accordance with local standard operating procedures for clinical sample types, and compliant with national standards for microbiology investigations [15]. These typically involve selective culture steps and species identification using MALDI-ToF (MALDI BioTyper, Bruker, Billerica, MA, USA).

Pseudo-anonymized extracts of all patient culture results and admission/discharge data covering the study period were obtained after the study was finished through the Infections in Oxfordshire Research Database to enable an evaluation of (i) baseline sampling denominators, (ii) the extent of relevant clinical isolate capture and (iii) the temporal and spatial overlap of any genetically related sequenced isolates from patients and sequenced isolates/metagenomes from sinks.

### Isolate sequencing and p-trap aspirate metagenomics

All isolates confirmed as *E. coli *, *K. pneumoniae* and *K. oxytoca* from patients and p-trap aspirates were extracted for sequencing using the QuickGene DNA extraction kit (Autogen, MA, USA) following manufacturer instructions, plus an additional mechanical lysis step prior to chemical lysis (FastPrep, MP Biomedicals, CA, USA; 6 m/s for two 40 s cycles).

For metagenomics, DNA was extracted from a subset of stored pellets (*n*=20) using the MoBio PowerSoil DNA isolation kit (Qiagen, Hilden, Germany) as per the manufacturer’s instructions, and including a mechanical lysis step of two 40 s cycles at 6 m/s in lysing matrix E and final elution in buffer CDT-1 (Autogen, MA, USA). In total, 45 ng of *Thermus thermophilus* DNA (reference strain HB27, ATCC BAA-163 [DSMZ, Germany]) was added to each sample in the PowerBead tube at the start of the experiment, prior to the addition of solution C1 as an internal control and normalization marker [11]. Sink aspirates were selected for metagenomic sequencing to enable evaluation of (i) microbiome differences within and between wards, (ii) longitudinal change in microbiota composition, and (iii) whether culture-negative sinks harboured the bacterial species being studied, i.e. indicating limited sensitivity of culture-based approaches.

Short-read sequencing (single isolate and metagenomics) was performed on the Illumina HiSeq 4000, pooling 192 isolate extracts and six metagenomes per lane, and generating 150 bp paired-end reads. Median metagenomic sequencing throughput was 547 Mb (IQR 499Mb to 942Mb) per sample.

### Computational methods

*Cultured isolate informatics*. Of the isolates sent for sequencing, 439/446 (98 %) sink and 46/46 (100 %) patient isolates were successfully sequenced and classified with Kraken/MiniKraken [16] as Enterobacterales, and used for subsequent analysis. Isolate consensus sequences were constructed by read mapping and consensus inference with respective *E. coli *, *K. oxytoca* and *K. pneumoniae* reference genomes AE014075.1, NC_018106.1 and CP000647.1 using Snippy 4.4.0 [17]. Isolate genomes were assembled using Shovill 1.0.4 [18]. Recombination-adjusted phylogenetic reconstruction was performed using runListCompare 0.3.8 [19] wrapping IQ-TREE 1.6.11 [20] and ClonalFrameML 1.12 [21]. Final core-genome alignments included 218/219 *E. coli* isolates, 165/167 *K *. *oxytoca* isolates and 98/99 *K *. *pneumoniae* isolates, all of which satisfied the runListCompare filtering criteria of perACGT_cutoff >=70 %, varsite_keep >=0.8 and seq_keep >=0.7. Overall, 100 SNP core-genome clusters were defined by single linkage clustering of runListCompare pairwise distance matrices. Trees were midpoint rooted prior to visualization. See supporting data repository for runListCompare configuration. Read-based MASH trees were constructed using MASH 2.2.2 [22] and RapidNJ 2.3.2 using 21 mers, a sketch size of 10 000 and a minimum abundance threshold of 10 *k*-mers. Nearest neighbours between sink and clinical isolates were identified in both Core SNP and MASH space. Assembly-based core and accessory genome partitioning was performed using PopPUNK 1.1.7 [23]. Resistance genotyping and phenotype prediction in cultured isolates was performed using ResPipe [11] and ARIBA 2.14.4 [24] with the CARD 3.0.3 database [25]. Tree comparisons (tanglegrams) were generated using the R package Dendextend 1.5.0 [26].

*Metagenome informatics*. Metagenomic sequences were analysed for taxonomic and antimicrobial resistance gene presence using ResPipe [11] and Kraken2 [27] with CARD database version 3.0.3. Large resistance gene families were clustered to facilitate visualization of resistance profiles (methodology documented in supporting data repository). A metagenomic assembly of the *mcr-4* gene was generated with MEGAHIT 1.2.9 [28], to which reads were aligned with Minimap2 2.17-r941 [29] and consensus inferred using Kindel [30]. Metagenomic summary statistics were generated using Pavian [31].

Data analysis was performed with the SciPy ecosystem [32] and JupyterLab [33]. Matplotlib [34], Bokeh and Microreact [35] were used for visualization.

## Results

### Diverse, often antimicrobial-resistant Enterobacterales lineages are frequent—and often persistent—colonizers of hospital sink drains

**Fig. 1.**
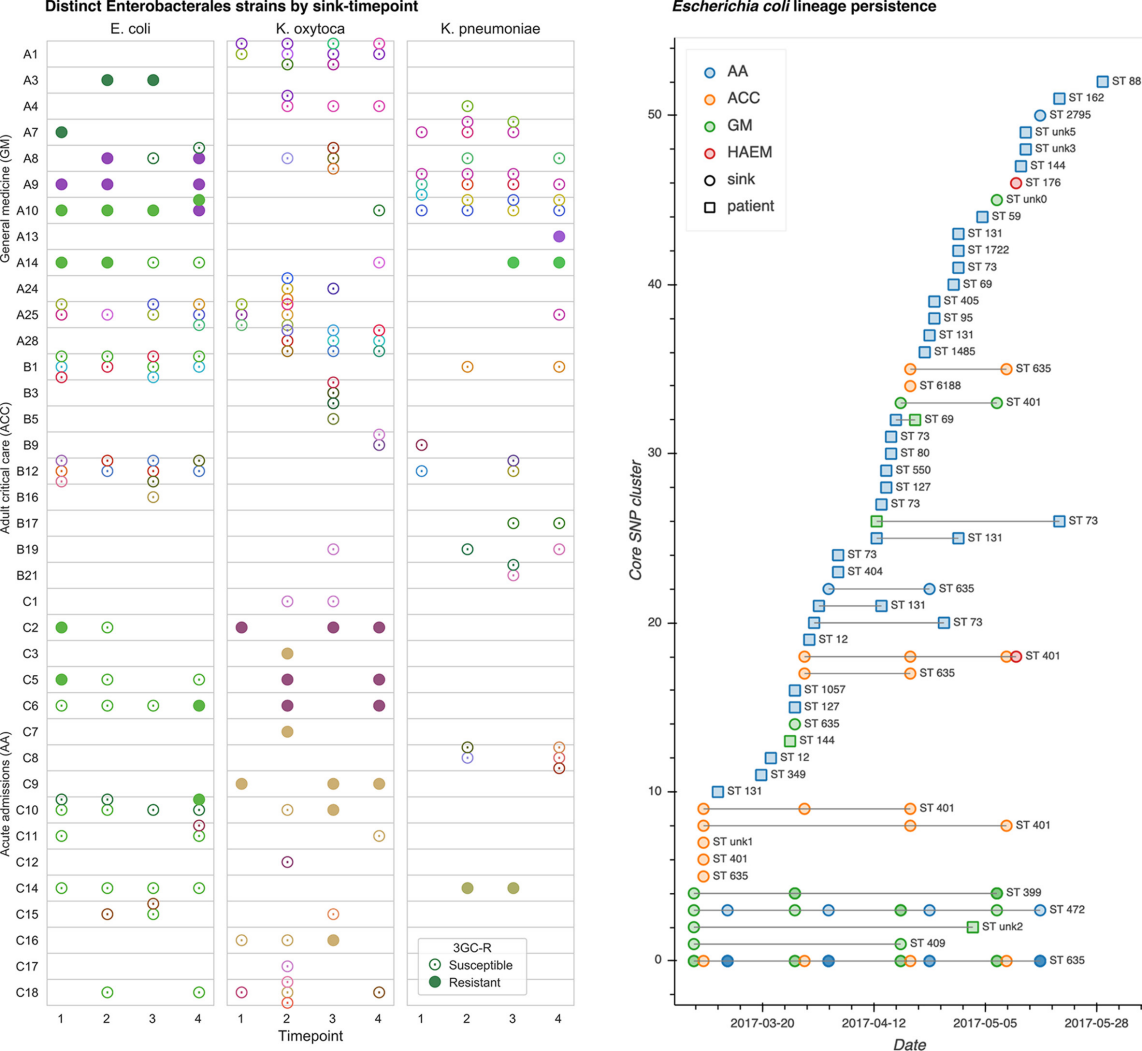
Cluster distribution and persistence. Left: strain-distinct cultured isolates of *E. coli *, *K. oxytoca* and *K. pneumoniae* from sink drain aspirates sampled over 12 weeks across three hospital wards. Different colours indicate distinct strains (defined by 100 SNP clusters), and cefpodoxime-resistant and/or selected ESBL-positive isolates are indicated by filled markers. Right: persistence of sink and contemporaneous patient *E. coli* strains throughout the sampling period.

In total, 439 Enterobacterales isolates comprising *E. coli* (*n*=180), *K. oxytoca* (*n*=166) and *K. pneumoniae* (*n*=93) were cultured and successfully sequenced at one or more timepoints from 12/20 (60 %), 9/23 (43 %) and 16/23 (70 %) sinks sampled four times over 12 weeks (March–May 2017) in general medicine (GM), adult critical care (ACC) and acute admissions (AA) wards, respectively (97/264 [37 %] sink-timepoints culture-positive overall; Fig. 1). A further 30 isolates of *E. coli* (*n*=13), *K. oxytoca* (*n*=13) and *K. pneumoniae* (*n*=4) were cultured from 11/59 (19 %) sinks in a haematology ward, sampled at a single timepoint only during this period (Fig. S1, available in the online version of this article). See Table S1 for surveyed sink descriptions. Species distributions (by culture) were relatively even across the general medicine ward, while the adult critical care unit was enriched for *E. coli *, and the acute admissions ward was depleted in *K. pneumoniae* (Table S2).

Analysis of whole-genome sequences from cultured Enterobacterales revealed widespread and sustained colonization of sinks by multiple sequence types (STs) of these species (Figs 1 and S1) In total, eight known and four novel *E. coli* STs were represented, of which STs 635 (*n*=109, 61 %), 401 (*n*=25, 14 %) and 472 (*n*=18, 10 %) accounted for 84 % of sequenced isolates (152/180). *Klebsiella* spp. STs were more varied: 15 known and six novel *K. oxytoca* STs were represented, of which the most frequent was ST177 (33/166, 20 %), while there were 18 known and one novel *K. pneumoniae* STs, the most frequent being ST872 (24/93, 26 %).

Across all locations and sink-timepoints, sequenced isolates comprised 20, 50 and 26 distinct lineages (defined as differing by <=100 recombination-adjusted core SNPs; see Methods) of *E. coli *, *K. oxytoca* and *K. pneumoniae,* respectively (Fig. 1, Table 1). Numerous sinks were colonized with multiple lineages (Figs 1, S1 and S2), reflecting significant diversity within species in sink niches. Of the 37 longitudinally sampled culture-positive sinks from which sequences were obtained, 31 (84 %) grew isolates of the same lineage across multiple timepoints, highlighting persistent background colonization illustrated for *E. coli* and *Klebsiella* spp. in respective Figs 1 and S3. Isolates resistant to third-generation cephalosporins were cultured at 16 sink-timepoints across 12 distinct sinks, with resistant and susceptible cultures of the same lineage co-occurring in 11/16 (69 %) sink-timepoints, suggestive of gain and/or loss of genes conferring cephalosporin resistance in this setting. No carbapenem-resistant isolates were cultured.

**Table 1.** Spatiotemporal distribution of 100 core SNP lineages of cultured *E. coli* (*n*=53), *K. oxytoca* (*n*=51) and *K. pneumoniae* (*n*=30) (a) overall, and (b) occurring in >1 isolate in sinks on wards that were repeatedly sampled

(a)		* E. coli *	* K. oxytoca *	* K. pneumoniae *	Total
**Lineages with >1 isolate**					
	Patient	28	1	3	32
	Sink	3	37	12	52
**Lineages with >1 isolate**					
Single timepoint	Same sink	5	6	3	14
	Different sinks; same ward	0	1	0	1
Multiple timepoints	Same sink	7	1	7	15
	Different sinks; same ward	1	4	4	9
	Different wards	3	1	0	4
Patients	Patient and sink (single timepoint)	1*	0	0	1
	Same patient	1	0	1	2
	Different patients	4	0	0	4
Total		53	51	30	134
**(b)**		***E. coli***	***K. oxytoca***	***K. pneumoniae***	**Total**
**Sink lineages with >1 isolate (excludes haematology ward**)					
Single timepoint	Same sink	5	4	3	12
	Different sinks; same ward	0	0	0	0
Multiple timepoints	Same sink	8	1	7	16
	Different sinks; same ward	1	4	4	9
	Different wards	2	1	0	3
Total		16	10	14	40

*Lineage has three isolates; all taken from the same ward; two from the same sink at the same timepointand one from a patient 2 months later.

### Enterobacterales can be highly abundant in sink drains, representing dominant populations in some wards

**Fig. 2.**
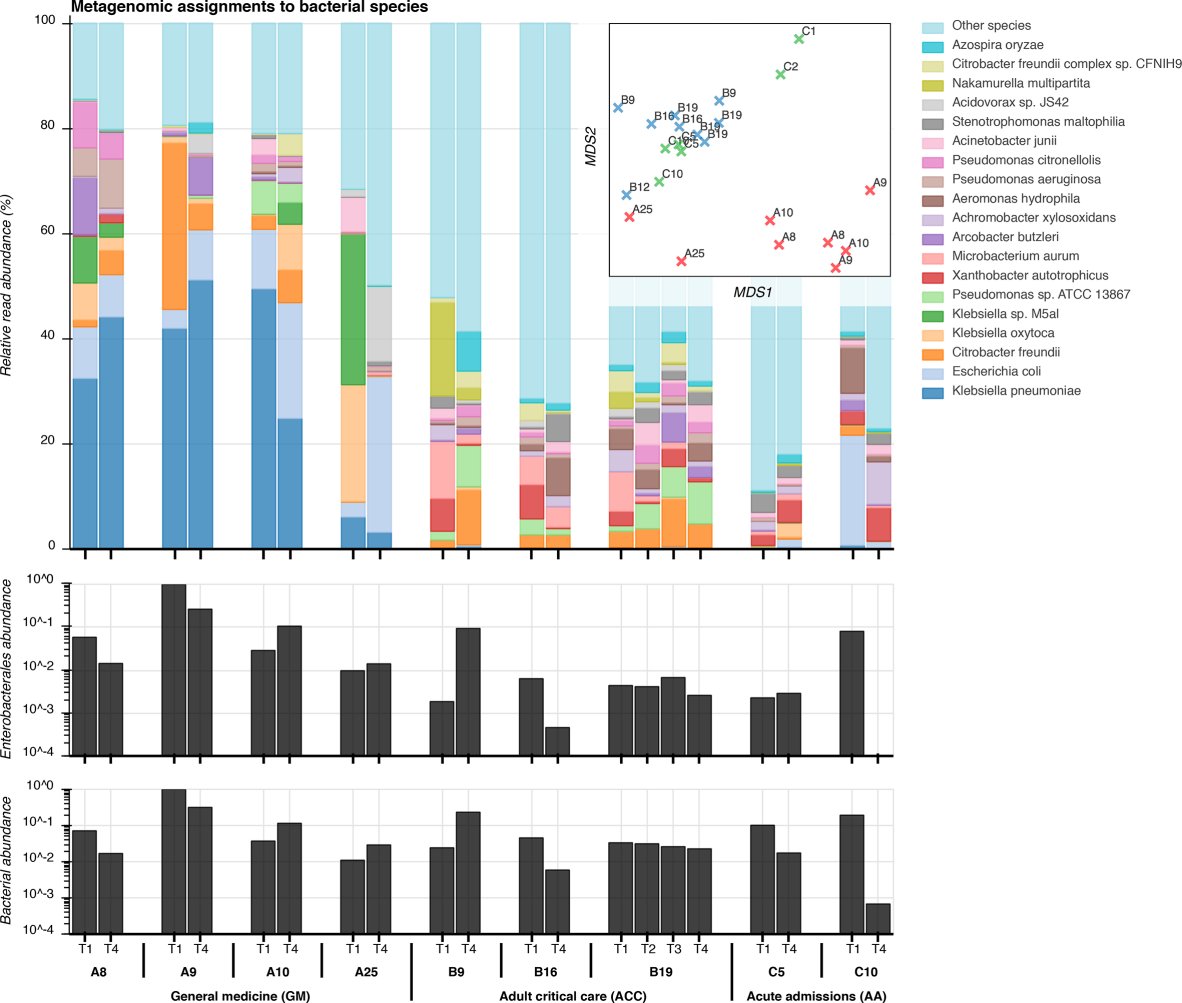
Taxonomic composition of sink microbiota from metagenomic sequencing. Top: relative abundance of the 20 most abundant bacterial species among sink drain aspirates (Kraken), inset with a corresponding multidimensional scaling (MDS) projection of pairwise distances between samples. Centre: spike-normalized relative abundance of species classifications at or below the order Enterobacterales among sink-timepoints. Bottom: spike-normalized relative abundance of Kraken classifications at or below the superkingdom bacteria.

Deep metagenomic Illumina sequencing was performed for 20 sink-timepoints on p-trap aspirates from seven sinks on the three wards at two timepoints, and all four timepoints for a single sink unit in the adult critical care ward (median throughput 547 Mb per sample; IQR 499Mb to 942Mb). The three most abundant bacterial genera were *Klebsiella *, *Escherichia* and *Citrobacter *, all common healthcare-associated pathogens (Fig. 2). Sink drains in the general medicine ward were the most abundantly colonized by Enterobacterales (Fig. 2), to which more than 50 % of reads were assigned, and were markedly less diverse than those in adult critical care and acute admissions wards, which had a dominance of *Klebsiella* spp., mirroring the culture results. 90 % of species-level classifications in the sink-timepoints from the general medicine ward came from a median of just 21 bacterial species, compared with medians of 310 and 450 species in the adult critical care and acute admissions wards, respectively. Microbial composition varied markedly between sampling timepoints for individual sinks, but sinks within wards exhibited more similar taxonomic profiles than those between wards (Fig. 2), suggesting distinct ward-based wastewater ecologies. Total metagenomic sequence content was hierarchically structured by ward and by sink (Fig. S4). Staff room sink A25 exhibited distinctive taxonomic and *k*-mer profiles from patient room sinks in the general medicine ward (Figs 2 and S4).

Sinks with high metagenomic abundance of the three Enterobacterales species reliably yielded corresponding cultures. The area beneath the receiver operating characteristic (ROC) curve for culture-based detection of these species was 0.93 (Fig. S5). When the relative metagenomic abundance of a species was above 0.1, 1 and 10 %, one or more cultures of the same organism were obtained in 58 % (18/31), 76 % (16/21) and 89 % (8/9) of sinks, respectively. Conversely, culture detection therefore failed in 42 % (13/31), 24 % (5/21) and 11 % (1/9) of cases where an Enterobacterales species was present at or above respective thresholds of 0.1, 1 and 10% metagenomic abundance. A single sink-timepoint (first sample from A8; general medicine) failed to culture any Enterobacterales, but yielded 4, 5 and 16% relative metagenomic abundances for the three study species. Thus metagenomic sequencing suggests that persistence may be even more widespread than indicated by culture alone.

### Most environmental Enterobacterales appear to cluster within specific sinks and wards, except for *E. coli* ST635, which is widely distributed

**Fig. 3.**
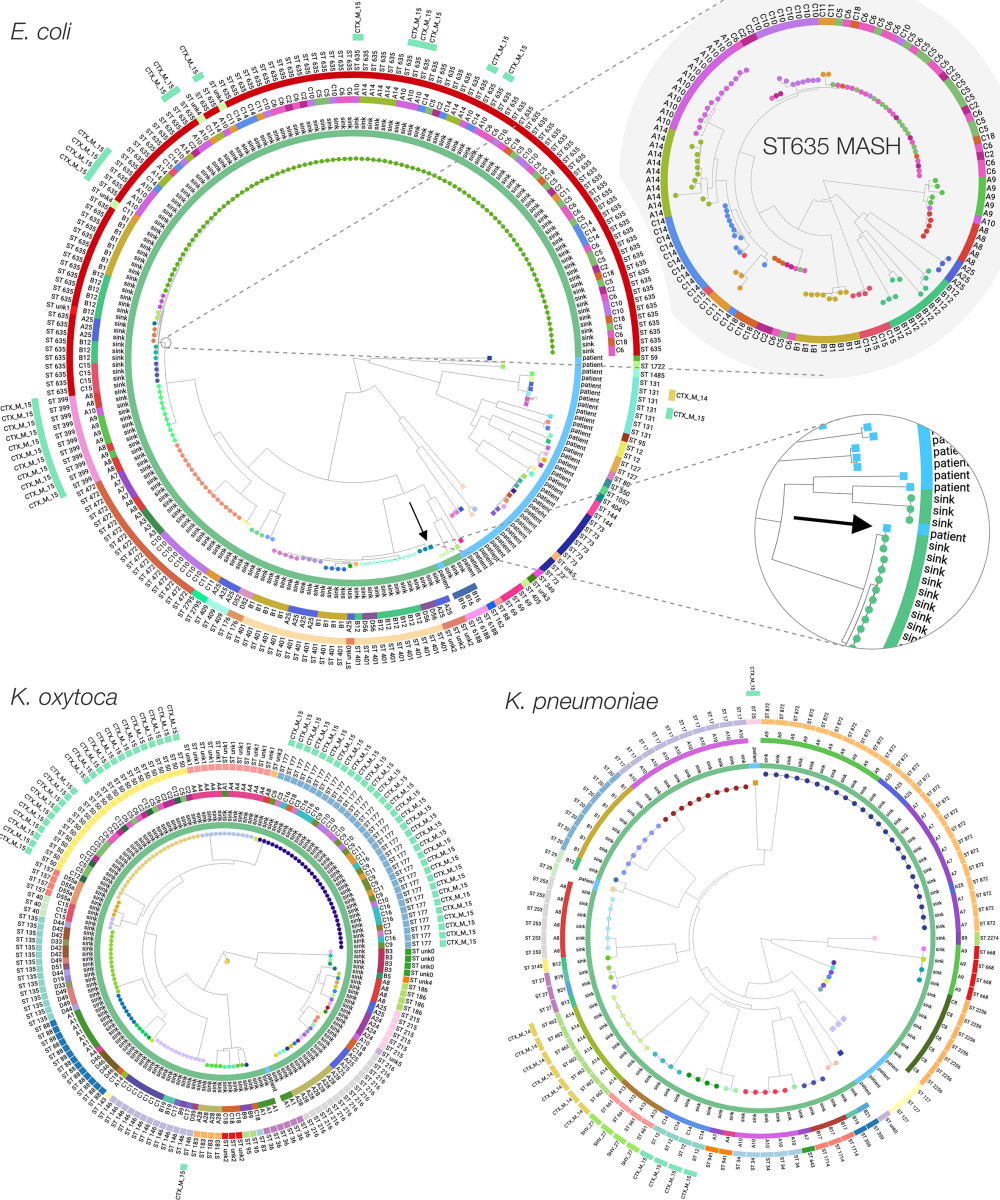
Maximum-likelihood phylogenies of *E. coli *, *K. oxytoca* and *K. pneumoniae* cultured from sink drain aspirates sampled over 12 weeks across three wards, with two enlargements corresponding to an *E. coli* ST635 neighbour-joining MASH subtree whose tips are coloured by sink, and genetic overlap between a sink culture and a urine culture from a patient with ward contact during the study. Tip colours indicate strains, with rings inside-to-out denoting: patient/sink, sink designation, sequence type, and ESBL genotype.

The 96 distinct 100 core SNP lineages found in sinks exhibited structure at the ward and sink level. Three were found in multiple wards (* E. coli* STs 472 and 635; *K. oxytoca* ST 146). Overall, 93 (97 %) lineages were only ever found in a single ward, and of the 39 lineages cultured twice or more, only 11 (28 %) were cultured from different sinks (Table 1). Further, of the 35 lineages cultured twice or more on wards which were repeatedly sampled (i.e. where it was theoretically possible for a lineage to be observed at different timepoints), 12 (34 %) were only seen at the same sink-timepoint, 13 (37 %) were seen in the same sink at different timepoints, and 10 (29 %) in different sinks at different timepoints. This structure was reflected in the recombination-adjusted core-genome species phylogenies (Fig. 3). The main exception to ward and sink-based clustering was *E. coli* ST635, which comprised more than half of isolates sequenced from sinks, and was found in 13/20 (65 %) *E. coli *-positive sinks.

However, there was sink-level clustering even within *E. coli* ST635, more clearly shown in neighbour-joining trees constructed from pairwise read-based MASH distances, representing both core and accessory genomic content (e.g. *E. coli* ST635 enlargement in Fig. 3; colours indicate distinct sinks). Although pairwise correlations between core and accessory genomic distances were high (Table S3), incorporating accessory content yielded additional resolution beyond core SNP distances (Fig. S6). Permutational analysis of variance (PERMANOVA) using pairwise core SNP and read-based MASH distances supported significant grouping of isolates from all three species by ward and by sink (*P*<0.001), most conclusively for *K. pneumoniae* (Table S4).

### Overall diversity among patient *E. coli* isolates was greater than among sink isolates, and included known high-risk clinical lineages

From March to May 2017, 1384 relevant clinical samples from 719 patients were submitted to the microbiology laboratory for processing (AA *n*=779, ACC *n*=365, GM *n*=240), of which 397/1384 (29 %) were positive for micro-organisms, and 115 were culture-positive for one of the study organisms (* E. coli* [*n*=104], *K. oxytoca* [*n*=2], *K. pneumoniae* [*n*=9]). In total, 46/115 (40 %) of these isolates were retrieved for sequencing, including 21/24 isolates from bloodstream infections, 3/6 from respiratory samples and 22/79 from urine samples; no isolates were retrieved for sequencing in six cases where study organisms were cultured from other clinical specimens (fluid, tissue, joint aspirates).

Among 39 sequenced *E. coli* patient isolates, 21 STs were represented, including known high-risk lineages (23/39 [59 %] isolates) not seen in sinks: namely ST73 (*n*=8), ST131 (*n*=7), ST69 (*n*=3), ST12 (*n*=2), ST127 (*n*=2) and ST95 (*n*=1). The single sequenced *K. oxytoca* isolate was ST36, and the six *K. pneumoniae* isolates came from four STs, including two high-risk lineages, ST25 and ST29. Across the three species, there were 34, 1 and 4 distinct lineages, respectively (Table 1).

### Genetic similarity of sequenced patient and environmental isolates

As well as being diverse, the 39 clinical *E. coli* isolates were phylogenetically distinct from most sink isolates, which largely came from just four sequence types (ST635, ST401, ST472 and ST399). The exception was an *E. coli* isolated from urine taken on the general medicine ward, which was 17 and 19 core SNPs from two nearest neighbour isolates from sink A25 in the same ward, sampled 58 days prior to the clinical sample (Fig. 1; right; cluster 4). A read-based MASH distance of 7×10^−6^ between this pair of isolates indicated very high total genomic (chromosome+accessory) similarity. A records search for admissions of this patient prior to commencing sink sampling revealed four inpatient admissions (of 0 [day case], 1, 2 and 5 nights’ duration), of which three included time on the acute admissions ward. There were no prior admissions onto the general medicine ward (in which their positive urine specimen was taken), and their 11-night spell on the general medicine ward commenced with a 7 h episode in acute admissions. The positive clinical specimen was taken 10 days after the patient’s admission onto the general medicine ward, indicating a large duration of exposure to a ward environment shown to be harbouring a very similar strain of *E. coli* to the patient’s urine culture. The next most closely related *E. coli* clinical isolate was 3688 core SNPs from its nearest sink neighbour (read-based MASH distance 0.009), reflecting the otherwise large evolutionary distances separating the cultured clinical and environmental *E. coli* (Fig. 3).

Unlike *E. coli *, the small numbers of *Klebsiella* spp. patient isolates were not phylogenetically segregated from environmental isolates, but the closest patient and sink isolates differed by 2558 core SNPs, indicating a lack of observed overlap over these timeframes.

Analysis of protein coding gene function using the Clusters of Orthologous Groups of proteins (COG) ontology highlighted functional differences between sink and patient isolates (Fig. S7). Accounting for the majority of clinical isolates, differences were most apparent among *E. coli *, in which sink and patient isolates were almost linearly separable in the first principle component (PC1; Fig. S7; right). Variable loadings for PC1 indicated COGs explaining the variation between sink and patient groups. The COG with highest loading was insertion sequence IS1R, highly abundant in sink *E. coli* isolates and possibly associated with the acquisition and expression of adaptive genes. Other highly explanatory COGs included genes involved in mobility, adhesion and iron metabolism, predicting functional adaptations of *E. coli* to the built environment of the sink (see supporting data repository).

### Antimicrobial resistance genes are prevalent and spatially structured with respect to sink location

**Fig. 4.**
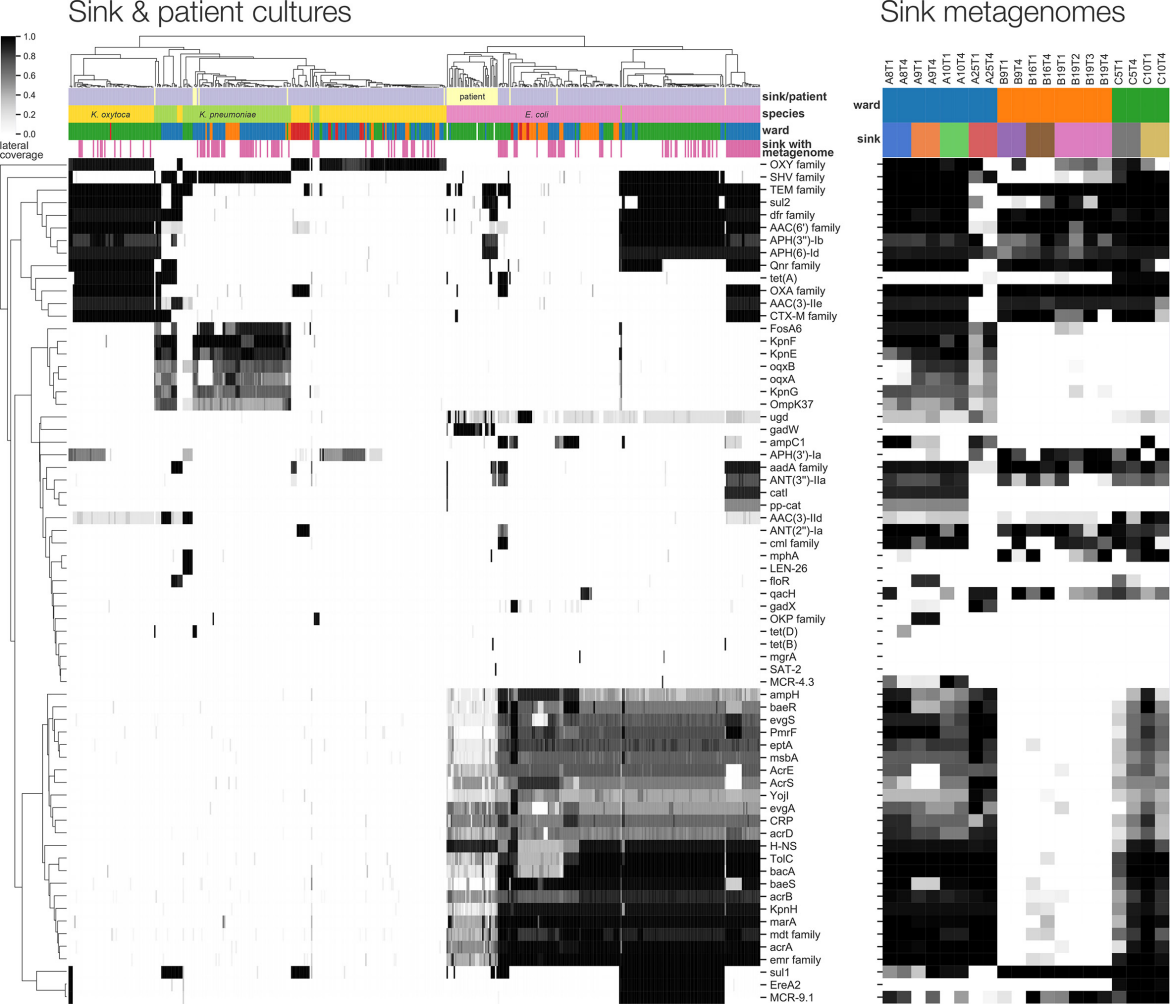
Antimicrobial resistance gene content of cultured isolates and sink drain metagenomes. Left: lateral coverage of ResPipe/CARD genes within sink drain and clinical isolates. Displayed genes attained >=75 % lateral coverage in one or more isolates. Right: corresponding lateral coverage of the same genes in sink drain aspirate metagenomes.

The presence of 571 clustered CARD antimicrobial resistance genes in cultured isolates was supported by >=75 % exactly matching read coverage reported by ResPipe (Fig. 4). Among these were known transmissible genes of clinical concern including beta lactamases (e.g. *bla*_TEM_, *bla*_CTX-M_, *bla*_SHV_), aminoglycoside resistance genes (*aac(3*), *aac(6*) families) and quinolone resistance genes (*qnr* family). Some of these, including *cmlA* and *qacH*, were widely seen in sink metagenomes but less frequently in cultured isolates, consistent with a background resistance reservoir that may pose a risk in different populations to those cultured (of either same or different species). Spatial structure with respect to sink location was evident among both cultured isolates and metagenomes, although resistance repertoires of isolates frequently clustered across ward boundaries, in keeping with findings of our prior core-genome analysis. Resistance genes detected in cultured sink isolates were also abundant within sink metagenomes at one or more timepoints. Sink drain metagenomes yielded 673 CARD genes exceeding 75 % coverage, and after clustering large gene families represented by many similar sequences (see Methods for detailed description), only five genes abundant in one or more cultured isolates were not detected in at least one metagenome. Notably, these five genes (*gadW*, *len-26*, *tet(B*), *mgrA* and *sat-2*) were all seen in isolates from sinks not subject to metagenomic sequencing, showing that resistance genes cultured from sink drains were highly contained in corresponding metagenomes.

Third-generation cephalosporin-resistant phenotypes in Enterobacterales sink isolates could be explained by the presence of major ESBL genes *bla*_SHV-27_, *bla*_CTX-M-14_ and *bla*_CTX-M-15_, detected by ARIBA/CARD in 4, 8 and 87 isolates, respectively. *bla*_CTX-M-15_ was identified in two distinct lineages of *E. coli* ST635 and ST399, restricted to three bay sinks (A8, A9, A10) in three adjacent rooms of the general medicine ward. *bla*_CTX-M-15_-positive *K. oxytoca* ST50 and ST177 were identified in 10 sinks (C2-3, C5-6, C7, C9-12, C16) on the acute admissions ward. *bla*_SHV-27_, *bla*_CTX-M-14_ and *bla*_CTX-M-15_ were observed in *K. pneumoniae* from sinks A13, A14 (general medicine) and C14 (acute admissions), respectively; one *K. pneumoniae* patient isolate was also *bla*_CTX-M-15_-positive. These findings suggest sink-associated isolates, such as *E. coli *, may represent reservoirs of clinically relevant resistance genes.

Surprisingly, the colistin resistance gene *mcr-4* was detected in the metagenomes—yet not cultured genomes—of three sinks in adjacent bays of the general medicine ward (A8-10). Assembly of the sink A10 metagenome generated a 5.4 kbp plasmid sequence containing an *mcr-4 gene* with 98.8 % overall identity at 94 % query coverage to an 8.7 kbp pMCR-4.2 plasmid previously reported in pigs from Italy, Spain and Belgium [36]. This *mcr* variant has been previously reported in European *Acinetobacter *, *Enterobacter *, *Salmonella* and *Escherichia* spp. but not to our knowledge in the UK. Screening all metagenomes for assembled *mcr-4* produced alignments in two sinks on the ward (A8, A9) across a total of six sink-timepoints, with coverage and abundance suggesting low and declining prevalence of this gene over time (Table S5). An *mcr-*positive *E. coli* (reported as *mcr4.3*) was cultured from sink C5 (acute admissions; second timepoint) according to both ARIBA/CARD and ResPipe/CARD. Metagenomic sequencing was performed for the first and the fourth but not the second timepoint aspirate for this particular sink. Another *mcr* gene, *mcr-9* was more widespread, and detected with complete coverage in 77 cultured isolates across 11 distinct sinks, predominantly but not exclusively in *E. coli* (73/77 occurrences). Plasmid replicon typing highlighted differences in plasmid presence between sink and clinical isolates, revealing 29 and 11 mutually exclusive replicon types to sink and patient isolates, respectively, and 15 types that were detected independently in both sink and patient isolates (Fig. S8, Table S6).

### Metagenomic screening suggests that clinical isolates may be more widely present in the environmental reservoir than observed from culture-based comparisons

**Fig. 5.**
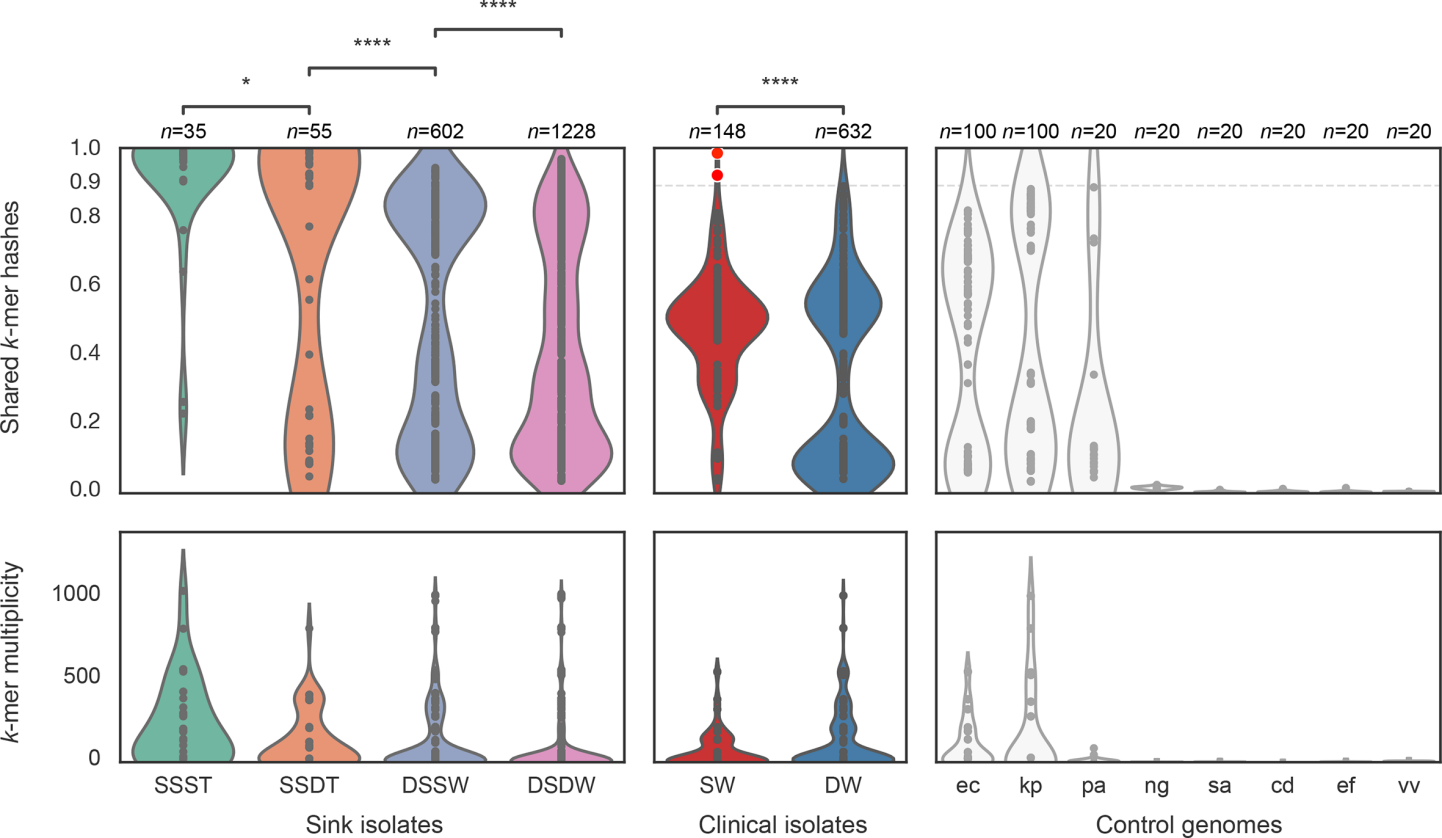
Metagenomic containment of sink (left) and patient (centre) cultured lineage-representative genome assemblies, and control genomes (right). Shared *k*-mer hashes and median *k*-mer multiplicity values are as reported by MASH Screen. SSST=same sink and same timepoint; SSDT=same sink at different timepoints (shared hashes Mann–Whitney *P*=0.013 vs. SSST); DSSW=different sinks of the same ward (*P*<0.0001 vs. SSDT); DSDW=sinks on a different ward (*P*<0.0001 vs. DSSW). SW=lineage-representative assemblies of clinical isolates in the same ward; DW=lineage-representative assemblies of clinical isolates from a different ward (*P*<0.0001 vs. SW). Control genomes comprised *E. coli *, *K. pneumoniae, P. aeruginosa*, *N. gonorrhoeae *, *S. aureus *, *C. difficile *, *E. faecalis* and *V. vulnificus *, shown abbreviated with binomial initials. A case of within-ward sink-patient overlap is highlighted with red markers, corresponding to high strain containment in the metagenomes of sink A25 timepoints 1 and 4.

Sink metagenomes were individually screened for *k*-mer containment of (i) lineage-representative sink isolate genome assemblies, (ii) lineage-representative patient isolate genome assemblies and (iii) core genomes of selected control organisms, including five clinical core genomes each from pathogenic strains of *E. coli* and *Klebsiella* spp. [37], together with NCBI canonical reference sequences for several pathogen species expected to be absent from sink drain microbiota (Fig. 5; left). This demonstrated similar sink, ward and temporal structure to that of culture, particularly underlying similarities in the microbiota of nearby sinks, as well as flux between sampling timepoints. Lineage-representative sink culture assemblies from the same sink and timepoint as the screened sink metagenome were the best contained, sharing the most *k*-mer hashes. Assembled isolates originating from the same sink at a different timepoint to the screened metagenome shared significantly fewer *k*-mer hashes than same sink/same timepoint comparisons (median [IQR]: 93 % [22.6 %–100.0%] vs. 99.6 % [97.7–100.0 %], Mann–Whitney *P*=0.013). The containment of lineage-representative assemblies from different sinks in the same ward as the metagenome was significantly lower still (49.2 % [13.8 %–84.8%] vs. 93.3 %[22.6 %–100.0%], *P*<0.0001), and so in turn were the remaining comparisons of cultures grown from different sinks in different wards to the screened metagenome (33.5 % [12.6 %–71.1%] vs. 49.2 % [13.8 %–84.8%], *P*<0.0001).

Among control genomes, *k*-mer hashes shared between sink metagenomes and the core genomes of *Neisseria gonorrhoeae *, *Staphylococcus aureus *, *Clostridioides difficile *, *Enterococcus faecalis* and *Vibrio vulnificus* did not exceed 3 %. Reference genomes of *E. coli *, *K. pneumoniae* and *Pseudomonas aeruginosa* were abundant and highly contained by many metagenomes, but none exceeded 90 % shared *k*-mer hashes (Fig. 5; right).

In contrast, screening for lineage-representative patient assemblies in sink metagenomes revealed significantly greater containment in terms of shared *k*-mer hashes (median [IQR]: 49 % [37 %–55%] vs. 32 % [8 %–56%], Mann–Whitney *P*<0.0001) between patient and environmental Enterobacterales collected from the same ward than from different wards (Fig. 5; centre), showing genetic similarity between clinical isolates from patients and uncultured isolates in sink niches in a given ward setting. Indeed, the only lineage-representative patient isolate with greater than 90 % sink metagenome *k*-mer containment was the *E. coli* urine culture described in the aforementioned case of sink-patient overlap, of which 99.5 and 93.0 % of *k*-mers were contained within the respective A25T1 (first timepoint) and A25T4 (fourth timepoint) sink metagenomes (Fig. 5; centre; red markers). Interestingly, this particular lineage was cultured at timepoint 1 but not timepoint 4 in sink A25, suggesting that culture may have failed to detect persistence of this lineage detected by metagenomics.

## Discussion

In this study, we have demonstrated that hospital sink drains are widely—and in many cases abundantly—contaminated with key Enterobacterales species causing healthcare-associated infections, and are potential reservoirs of multiple resistance genes encoding resistance to important clinical antimicrobials. Populations of antimicrobial-resistant and susceptible *E. coli* and *Klebsiella* spp. may be persistent colonizers of sinks, and different wards may have markedly different sink ecosystems, perhaps reflecting different and potentially modifiable infrastructures, selection pressures and contributing sources. Ward and sink-level genetic structure was especially evident within the accessory genome, and reflected in the observed repertoires of resistance genes. Nevertheless, a handful of key Enterobacterales lineages persisted across multiple wards throughout the duration of the sampling period, and sinks across ward settings were common reservoirs of important clinical resistance genes, including those encoding resistance to beta-lactams, sulphonamides, aminoglycosides and quinolones. Characterizing these highly diverse reservoirs is difficult, and we have shown that combination approaches utilizing metagenomics and sequencing of cultured isolates are complementary in understanding the diversity of species, lineages and the resistance genes present within these niches. For example, metagenomics highlighted several cases of abundant *mcr*-4 in sink drain aspirates from which cultured Enterobacterales isolates did not carry the gene. Furthermore, in the case of an *E. coli* lineage overlapping between sink and patient, metagenomics supported a hypothesis of persistent colonization throughout the sampling period, whereas culture detected this lineage only once.

Colonization patterns of sink niches differed markedly between the two genera investigated. *E. coli* strains have evolved to colonise and adapt to multiple niches, including some which have adopted pathogenic lifestyles, and appear to have different distributions in humans, domesticated and wild animals and the environment. There is however no absolute correlation between phylogenetic lineage and any given niche, and overlaps are observed. Interestingly, in our study, more than half of the *E. coli* sink isolates cultured were ST635, which has been recently described as a highly adapted, resistance- and virulence gene-enriched wastewater-associated strain thought to be globally distributed, but is also found in humans, animals and other environments [38]. Of note, it has been observed in association with several clinically relevant transmissible resistance genes, including ESBLs, carbapenemases and rRNA methylases, and was one of only two *E. coli* STs in our study that harboured an ESBL (*bla*_CTX-M-15_). We observed presence/absence of *bla*_CTX-M-15_ across closely related ST635 isolates, suggesting that this gene may be frequently lost/gained in sinks. Also notable in the context of ST635 was the ability of read-based *k*-mer composition to resolve fine-grained structure between the populations of different sinks, beyond that observed in the core-only SNP phylogeny. Other common *E. coli* sink lineages were ST399 and ST472, which to date have predominantly been seen in humans/animals, rather than the environment.

The phylogenetic distribution of sink isolates of *K. pneumoniae* appeared to mirror that seen in a global collection of isolates [39], providing little evidence that a particular lineage was predominating in, or particularly adapted to, the wastewater environment. Studies of the population structure of unselected *K. oxytoca* are limited, but again we observed a diverse population amongst sink isolates, with a deep branch separating two distinct groups as previously described. Interestingly, two *K. oxytoca* lineages associated with *bla*_CTX-M-15_ were widely distributed amongst sinks in the acute admissions ward; outbreaks of ESBL- and carbapenem-associated *K. oxytoca* in association with contaminated handwashing sinks have been described in other settings [40].

Genomic overlap with sink isolates was identified in 1/46 (2 %; 95 % CI: 0.05–11 %) of all sequenced isolates causing clinical infections over the same timeframe, with a temporal association consistent with acquisition from a sink source (i.e. sink isolate observed first), and following 10 days of patient exposure to a ward environment wherein the overlapping strain was previously cultured. We may have significantly underestimated the degree of overlap between these two compartments for several reasons. Firstly, we have shown the diversity in sink niches is substantial, and with a culture-based approach agnostic to any selective marker, even sequencing 444 isolates from 48 sinks will have limited ability to capture the underlying diversity for complete comparison of sink-patient pairs at the isolate-level. Supporting this, screening the metagenomes of a subset of 20 sinks using patient isolates suggests that overlap between these reservoirs may be more common than observed at the isolate-level. Second, clinical isolates represent the tip of the iceberg of any transmission chain, with the majority of transmission events likely occurring between gastrointestinal tract (asymptomatic carriage) and the wastewater environment. Nonetheless, in the context of understanding how sinks may be contributing to infection caused by *Klebsiella* spp. and *E. coli *, focusing on clinical isolates seems appropriate. Third, the interval between sampling dates for our observed patient-sink isolate-pair was 58 days, suggesting that the timeframe between acquisition from the environment and infection may be long, and may not be adequately captured with a study timeframe spanning 3 months. Refining estimates of the risk of patient infection associated with colonized sink sites ideally requires larger studies, over longer timeframes, and which ideally also capture transmission events resulting in gastrointestinal colonisation as an intermediate step to invasive infection.

In addition, a major study limitation is the fact that only 46/107 patient isolates could be successfully retrieved (due to the high turnover of samples in our high-volume service laboratory), and *Klebsiella* spp. cultures were especially limited. The risks of transmission and possibly sink-associated infection could be more clearly defined by more extensive sampling over a greater timeframe, and thorough investigation into the exchange of resistance-associated mobile genetic elements by, for example, using long-read sequencing to reconstruct plasmid sequences [41, 42]. Doing so would require a considerable increment in resource. Characterizing microbial diversity present on sink strainers would also be of benefit, as the risks of droplet-mediated dispersal from sink drains have been shown to be most significant when the sink drain is located immediately below the tap, and if the organisms migrate from the sink trap onto the strainer [43, 44]. However, given the different sink structures across wards, the p-trap was the only site that could be consistently sampled (since ACC had horizontally draining sinks without strainers). Characterizing factors that might be associated with greater predominance of Enterobacterales and drug-resistant Enterobacterales, such as sink usage, ward-level antimicrobial usage and patient populations, would also be of interest.

In conclusion, without conditioning on the presence of resistance markers, we have demonstrated that colonization of ward sink drains with diverse and abundant populations of Enterobacterales, including drug-resistant lineages, is common and persistent. The evidence linking contaminated, unmitigated wastewater reservoirs (including sink drains) in healthcare settings with outbreaks of colonization/disease with drug-resistant Gram-negative bacilli in patients seems clear [5, 45], but no study to our knowledge has focused on the potential risk posed by Enterobacterales in sinks in general. Screening of sinks is not carried out in the absence of observed outbreaks, making it difficult to quantify wider patient-associated risk from the studies available. We demonstrate that contaminated sinks may be contributing to a proportion of healthcare-associated infections caused by Enterobacterales, and further work to investigate how to reduce the risk posed by this hospital environmental reservoir is warranted.

## Data Bibliography

1. Chau KK, Constantinides B, and Stoesser N. Sink drain isolate genomic sequences: Enterobacterales colonisation of hospital sink drains. https://www.ncbi.nlm.nih.gov/bioproject/PRJNA604910. (2020)

2. Constantinides B, Lipworth S, Quan TP, and Stoesser N. Clinical isolate genomic sequences: Gram-negative bacteremias in Oxfordshire. https://www.ncbi.nlm.nih.gov/bioproject/PRJNA604975. (2020)

3. Constantinides B, Chau KK, and Stoesser N. Sink drain metagenomic sequences: Surveillance of Escherichia coli and Klebsiella spp. in hospital sink drains. https://www.ebi.ac.uk/ena/data/view/PRJEB36775. (2020)

## Supplementary Data

Supplementary material 1
