# Supplementary material for: Genomic surveillance of Escherichia coli and Klebsiella spp. in hospital sink drains and patients
Source: Microb Genom. 2020 Jun 18;6(7):mgen000391. doi: 10.1099/mgen.0.000391 (PMC7478627; doi:10.1099/mgen.0.000391)
Supplement: Supplementary material 1 [file mgen-6-391-s001.pdf]

			Timepoint			
Sink	Ward	Sink location	1	2	3	4
A1	General medicine	Staff toilet (entrance)	positive	positive	positive	positive
A3	General medicine	Patient sideroom 1 (1 bed)	negative	positive	positive	negative
A4	General medicine	Patient sideroom 2 (1 bed)	negative	positive	positive	positive
A5	General medicine	Patient toilet (entrance)	negative	negative	negative	negative
A7	General medicine	Patient bay 1 (4 beds)	positive	positive	positive	negative
A8	General medicine	Patient bay 2 (4 beds)	negative	positive	positive	positive
A9	General medicine	Patient bay 3 (4 beds)	positive	positive	positive	positive
A10	General medicine	Patient bay 4 (4 beds)	positive	positive	positive	positive
A11	General medicine	Patient toilet	negative	negative	negative	negative
A13	General medicine	Patient sideroom 3 (1 bed)	negative	negative	negative	positive
A14	General medicine	Patient sideroom 4 (1 bed)	positive	positive	positive	positive
A15	General medicine	Patient toilet 1	negative	negative	negative	negative
A18	General medicine	Patient toilet 2	negative	negative	negative	negative
A21	General medicine	Patient toilet 3	negative	negative	negative	negative
A23	General medicine	Medicine preparation room	negative	negative	negative	negative
A24	General medicine	Reception area	negative	positive	positive	negative
A25	General medicine	Staff room	positive	positive	positive	positive
A26	General medicine	Sluice room	negative	negative	negative	negative
A27	General medicine	Sluice room	negative	negative	negative	negative
A28	General medicine	Patient room sideroom 5 (1 bed)	negative	positive	positive	positive
B1	Acute critical care	Relatives' day room	positive	positive	positive	positive
B2	Acute critical care	Relatives' toilet	negative	negative	negative	negative
B3	Acute critical care	Staff toilet M	negative	negative	positive	negative
B4	Acute critical care	Staff toilet F	negative	negative	negative	negative
B5	Acute critical care	Patient sideroom 1 (1 bed)	negative	negative	positive	negative
B6	Acute critical care	Patient bay 1 (6 beds)	negative	negative	negative	negative
B7	Acute critical care	Patient sideroom 2 (1 bed)	negative	negative	negative	positive
B8	Acute critical care	Patient bay 1 (6 beds)	negative	negative	negative	negative
B9	Acute critical care	Patient bay 1 (6 beds)	positive	negative	negative	positive
B10	Acute critical care	Laboratory	negative	negative	negative	negative
B11	Acute critical care	Laboratory	negative	negative	negative	negative
B12	Acute critical care	Staff room kitchen sink	positive	positive	positive	positive
B13	Acute critical care	Sluice room	negative	negative	negative	negative
B14	Acute critical care	Sluice room	negative	negative	negative	negative
B15	Acute critical care	Sluice room	negative	negative	negative	negative
B16	Acute critical care	Patient bay 2 (4 beds)	negative	negative	positive	negative
B17	Acute critical care	Patient bay 2 (4 beds)	negative	negative	positive	positive
B18	Acute critical care	Patient bay 2 (4 beds)	negative	negative	negative	negative
B19	Acute critical care	Patient bay 3 (4 beds)	negative	positive	positive	positive
B20	Acute critical care	Patient bay 3 (4 beds)	negative	negative	negative	negative
B21	Acute critical care	Patient sideroom 3 (1 bed)	positive	negative	positive	negative
B22	Acute critical care	Patient sideroom 4 (1 bed)	negative	negative	negative	negative
B23	Acute critical care	Dirty utility sink	negative	negative	negative	negative
C1	Acute admissions	Staff toilet	negative	positive	positive	negative
C2	Acute admissions	Patient sideroom 1 (1 bed)	positive	positive	positive	positive
C3	Acute admissions	Female patient toilet	negative	positive	negative	negative
C4	Acute admissions	Female patient toilet and shower	negative	negative	negative	negative
C5	Acute admissions	Patient bay 1 (6 beds)	positive	positive	negative	positive
C6	Acute admissions	Patient bay 2 (4 beds)	positive	positive	positive	positive
C7	Acute admissions	Patient sideroom 2 (1 bed)	negative	positive	negative	negative
C8	Acute admissions	Patient sideroom 3 (1 bed)	negative	positive	negative	positive
C9	Acute admissions	Patient sideroom 4 (1 bed)	positive	negative	positive	positive
C10	Acute admissions	Patient bay 3 (4 beds)	positive	positive	positive	positive
C11	Acute admissions	Patient bay 4 (4 beds)	positive	negative	negative	positive
C12	Acute admissions	Patient sideroom 5 (1 bed)	negative	positive	negative	negative
C13	Acute admissions	Patient sideroom 6 (1 bed)	negative	negative	negative	negative
C14	Acute admissions	Patient bay 5 (7 beds)	positive	positive	positive	positive
C15	Acute admissions	Patient bay 5 (7 beds)	negative	positive	positive	negative
C16	Acute admissions	Near-patient testing room	positive	positive	positive	negative
C17	Acute admissions	Patient toilet waiting room	negative	positive	negative	negative
C18	Acute admissions	Ambulatory bay	positive	positive	negative	negative
C19	Acute admissions	Male patient toilet	negative	negative	negative	negative
C20	Acute admissions	Male patient toilet and shower	negative	negative	negative	negative
C21	Acute admissions	Dirty utility sink	negative	negative	negative	negative
C22	Acute admissions	Patient toilet	negative	negative	negative	negative
C23	Acute admissions	Treatment/medicines room	negative	negative	negative	negative

TABLE S1. Surveyed sinks

ward	species	count	ward sum	%
GM	*E. coli*	59	179	33%
	K. oxytoca	56	179	31%
	K. pneumoniae	64	179	36%
ACC	*E. coli*	36	64	56%
	K. oxytoca	10	64	16%
	K. pneumoniae	18	64	28%
AA	*E. coli*	79	166	48%
	K. oxytoca	76	166	46%
	K. pneumoniae	11	166	7%
HAEM	*E. coli*	6	30	20%
	K. oxytoca	24	30	80%
	K. pneumoniae	0	30	0%

TABLE S2. Cultured Enterobacterales by ward. The distribution of cultured Enterobacterales target species by ward

Species	Pairwise distance comparison		Mantel *r*	*p*
E. coli	reads-core-snp	reads-mash	0.879	0.001
		assemblies-core-mash	0.987	0.001
		assemblies-accessory-mash	0.892	0.001
	reads-mash	assemblies-core-mash	0.893	0.001
		assemblies-accessory-mash	0.870	0.001
	assemblies-core-mash	assemblies-accessory-mash	0.933	0.001
K. oxytoca	reads-core-snp	reads-mash	0.865	0.001
		assemblies-core-mash	0.901	0.001
		assemblies-accessory-mash	0.857	0.001
	reads-mash	assemblies-core-mash	0.995	0.001
		assemblies-accessory-mash	0.951	0.001
	assemblies-core-mash	assemblies-accessory-mash	0.938	0.001
K. pneumoniae	reads-core-snp	reads-mash	0.808	0.001
		assemblies-core-mash	0.800	0.001
		assemblies-accessory-mash	0.891	0.001
	reads-mash	assemblies-core-mash	0.996	0.001
		assemblies-accessory-mash	0.942	0.001
	assemblies-core-mash	assemblies-accessory-mash	0.916	0.001

TABLE S3. Pairwise Mantel correlation of different within-species distance matrices. These include recombination-adjusted core SNP phylogeny (reads-core-snp), read-based MASH distance (reads-mash) and PopPUNK estimates of core and accessory genomic distance from *de novo* assemblies (assemblies-core-mash, assemblies-accessory-mash).

			PERMANOVA		PERMDISP	
Factor	Species	Distances	Pseudo-F	*p*	Pseudo-F	*p*
Sink	*E. coli*	assemblies-acc-mash	11.74	0.001	11.67	0.001
		assemblies-core-mash	8.31	0.001	10.52	0.001
		core-snp	7.21	0.001	5.68	0.001
		reads-mash	6.30	0.001	5.45	0.001
	K. oxytoca	assemblies-acc-mash	9.61	0.001	3.05	0.001
		assemblies-core-mash	10.62	0.001	2.53	0.001
		core-snp	12.85	0.001	2.01	0.001
		reads-mash	9.53	0.001	2.66	0.001
	K. pneumoniae	**assemblies-acc-mash**	**13.03**	0.001	1.56	0.133
		**assemblies-core-mash**	**9.65**	0.001	1.49	0.084
		**core-snp**	**9.51**	0.001	1.38	0.337
		**reads-mash**	**10.30**	0.001	1.54	0.078
Ward	*E. coli*	assemblies-acc-mash	20.98	0.001	23.16	0.001
		assemblies-core-mash	18.16	0.001	29.86	0.001
		core-snp	25.48	0.001	51.71	0.001
		reads-mash	12.57	0.001	12.45	0.001
	K. oxytoca	assemblies-acc-mash	18.71	0.001	47.19	0.001
		assemblies-core-mash	21.54	0.001	86.31	0.001
		core-snp	24.59	0.001	43.62	0.001
		reads-mash	20.86	0.001	83.74	0.001
	K. pneumoniae	**assemblies-acc-mash**	**12.15**	0.001	0.45	0.656
		**assemblies-core-mash**	**12.40**	0.001	1.17	0.307
		**core-snp**	**7.53**	0.001	0.99	0.407
		**reads-mash**	**13.32**	0.001	1.62	0.185

TABLE S4. Permutational analysis of variance. Permutation tests for association of genetic structure with ward (n=3) and sink (n=18) for three species of sink drain Enterobacterales. Corresponding test results are shown for differential dispersion between groups (PERMDISP). Bold type indicates significant ($p<0.05$) group association under PERMANOVA in the absence of significant differential dispersion (PERMDISP).

	mcr-4 gene coverage (%)	mean depth
sink-timepoint		
A10T1	100.0	31.6
A10T4	92.4	2.7
A8T1	73.1	1.1
A8T4	14.2	0.2
A9T1	51.5	0.6
A9T4	32.2	0.4

TABLE S5. *mcr-4* coverage. Sequencing coverage and mean depth of the 1,626bp metagenome-assembled *mcr-4* gene from sink A10, to which metagenomic short reads mapped from three sinks (including A10) across six sink-timepoints within the general medicine ward.

Replicon type	Frequency	Presence
IncFIB_K_-	331	sink and patient
IncFII_K_	185	sink and patient
IncFI-	129	sink and patient
p0111	127	sink and patient
Col440I	121	sink only
IncFII_-	118	sink only
FIA_pBK30683_+	103	sink and patient
IncHI2	102	sink only
IncHI2A	102	sink only
FII_pBK30683_	87	sink only
IncFII_pKP91_	80	sink only
Col_pHAD28_	77	sink only
IncFII_S_	41	sink only
ColRNAI	40	sink and patient
IncR	35	sink only
IncFIB_AP001918_	28	sink and patient
IncFIB_pKPHS1_	23	sink and patient
pKPC_CAV1321	22	sink only
IncHI1B_-	19	sink only
IncHI1A	18	sink only
Col156	17	patient only
Col_IRGK_	17	sink only
IncY	16	sink and patient
IncFIB_pNDM_Mar_	15	sink and patient
Col_IMGS31_	15	sink only
IncHI1B_pNDM_MAR_	13	sink and patient
Inc-_1	13	sink only
repA_pKOX_	12	sink only
IncM1	11	sink only
IncFIB_pQil_	11	sink only
Col_MG828_	10	patient only
IncB_O_K_Z	10	patient only
IncFIA	9	sink and patient
pKPC_CAV1320	8	sink only
IncQ1	8	sink only
Col_MGD2_	8	sink only
IncX5_1	7	sink only
IncFII_p-	7	patient only
IncFII_pCRY_	5	sink only
IncI-	4	sink and patient
IncX1	4	sink and patient
IncN	4	sink only
IncI2-	3	patient only
Col8282	3	patient only
IncX1_1	3	sink and patient
Inc-	3	sink only
pESA2	3	sink only
pSL483+	3	sink only
Col_BS512_	2	patient only
Col_MP18_	2	patient only
IncHI1A_NDM_CIT_	1	sink only
Col440II	1	sink only
Col_KPHS6_	1	patient only
IncX4	1	patient only
IncFIB_H89_PhagePlasmid_	1	patient only

TABLE S6. Clustered plasmid replicon types mutually exclusive to sink and patient isolates alongside corresponding frequency, generated using ARIBA and the PlasmidFinder database.

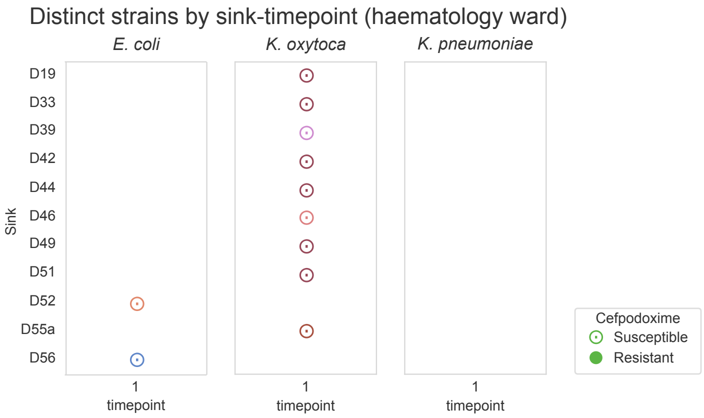

FIGURE S1. Cultured strains observed on the haematology ward. Different colours indicate distinct 100 core SNP strains, and cefpodoxime-resistant and/or ESBL gene-positive isolates are indicated by filled markers.

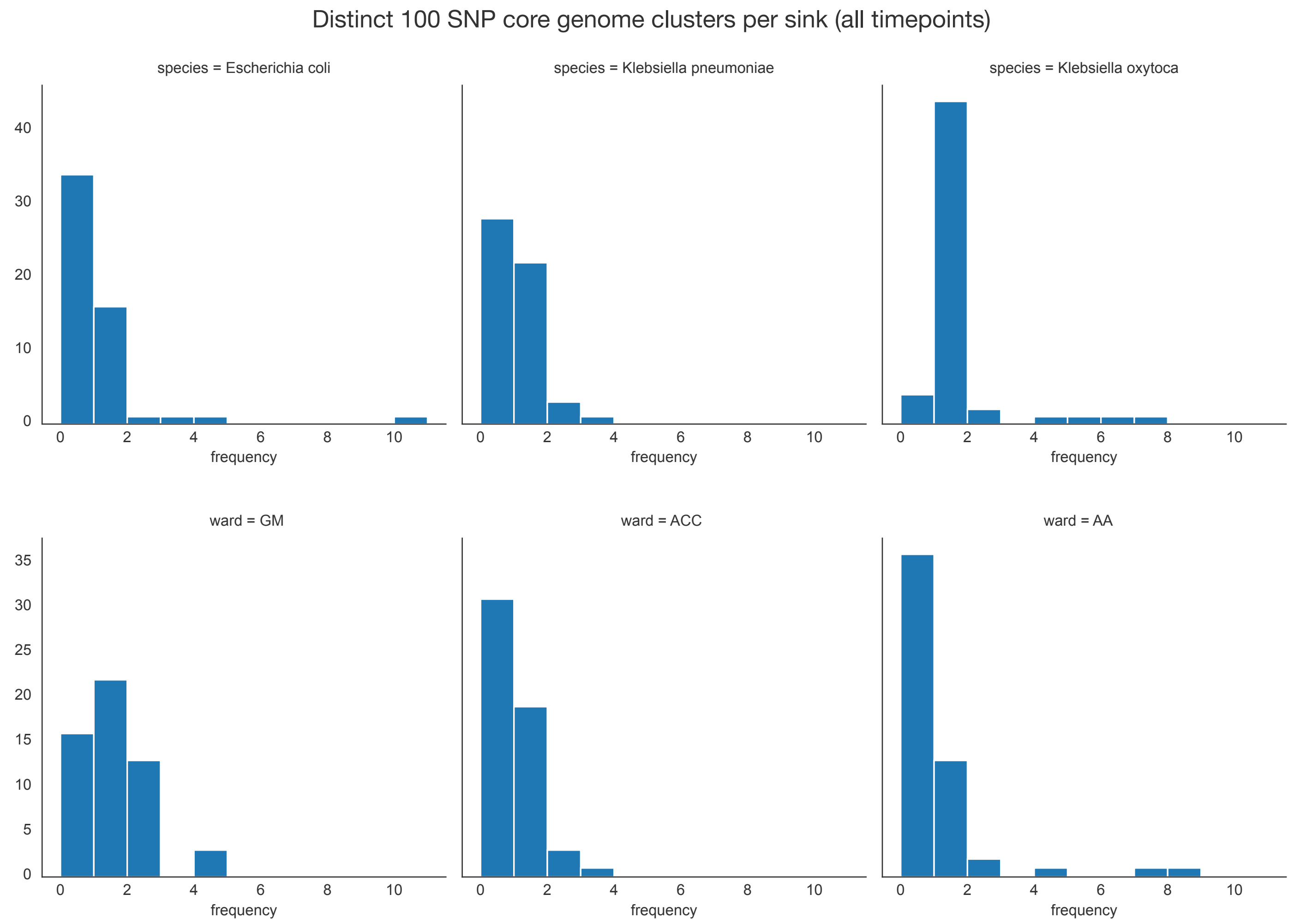

FIGURE S2. Distinct core genome SNP clusters cultured per sink by ward and species.

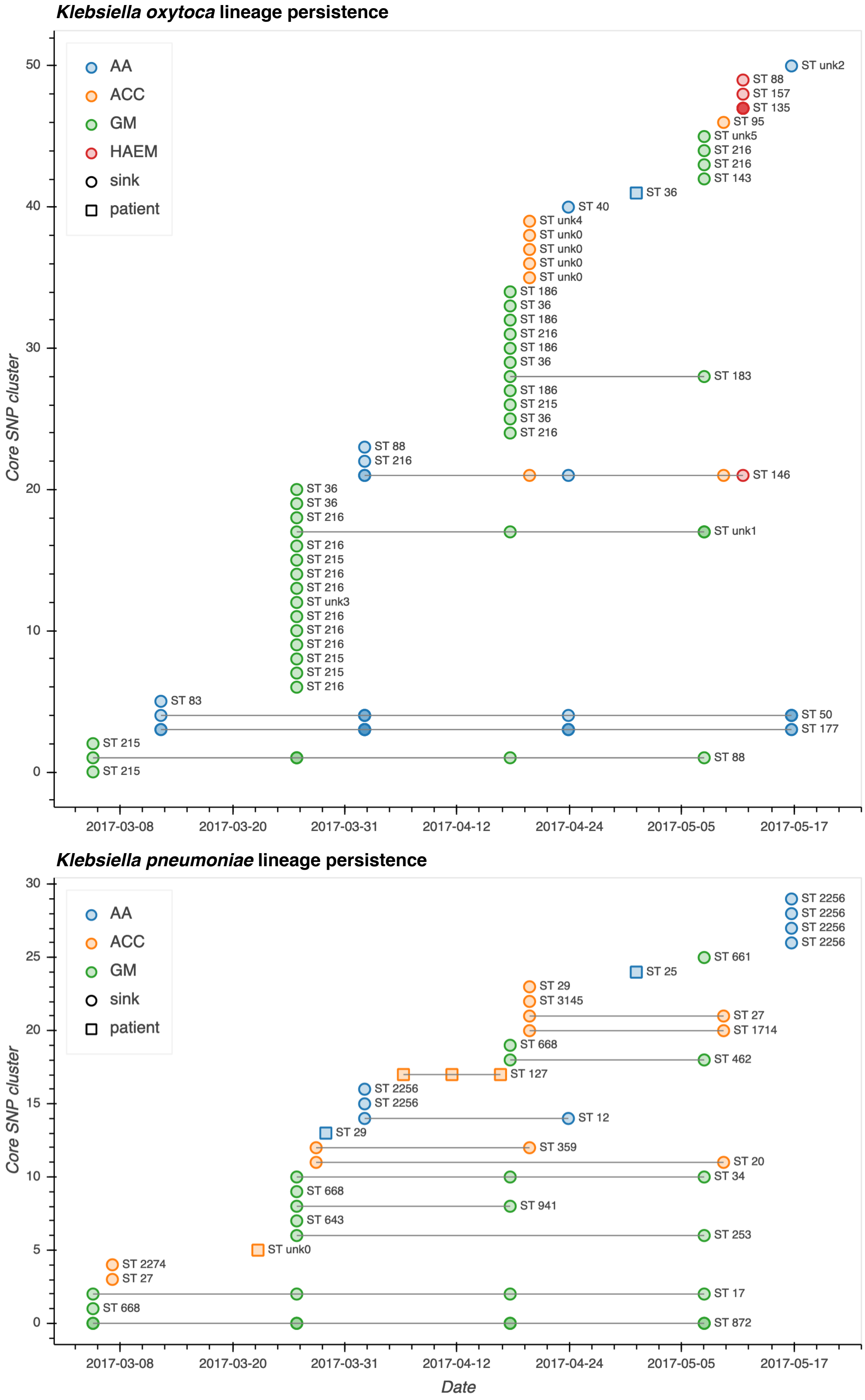

FIGURE S3. ***Klebsiella*** **spp. lineage persistence in cultured sink drain aspirates and contemporaneous clinical isolates from patients with ward contact during the sampling period.**

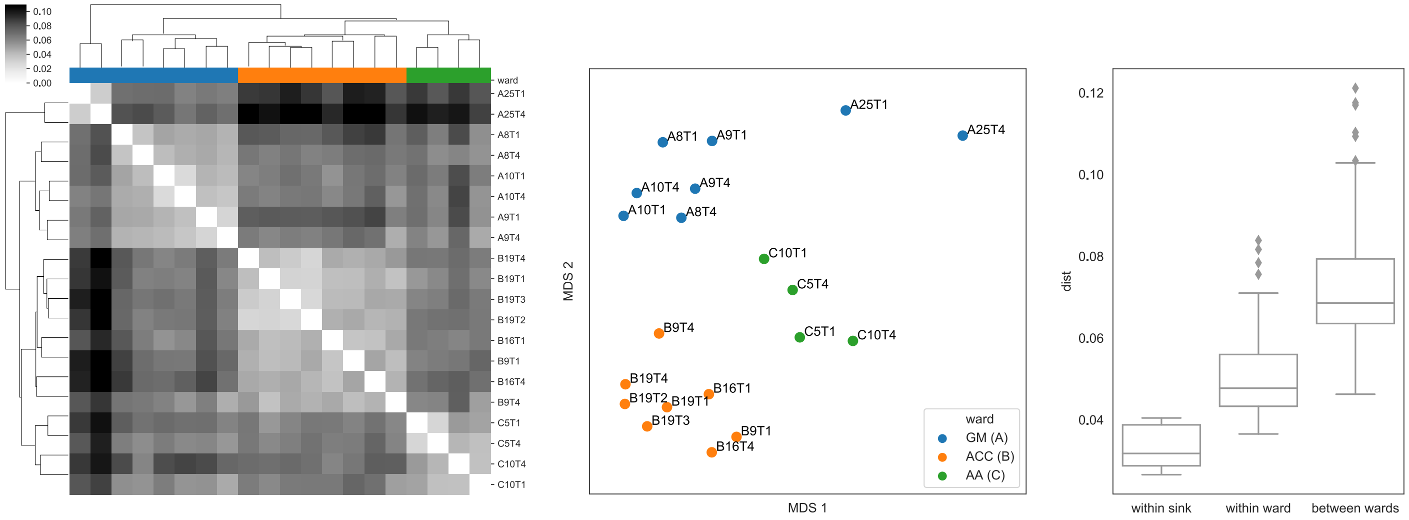

FIGURE S4. Spatial structure of sink metagenome *k*-mer composition. Left and centre: visualisation of 31mer pairwise MASH distances of total metagenome content using hierarchical clustering (left) and multidimensional scaling (centre). Right: comparison of within sink, within ward and between ward pairwise MASH distances.

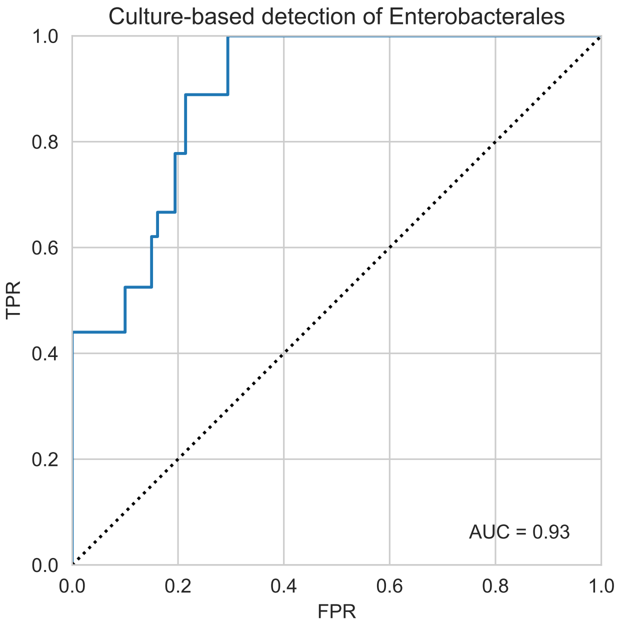

FIGURE S5. Receiver operating characteristic (ROC) for detection of Enterobacterales by culture with varying metagenomic abundance.

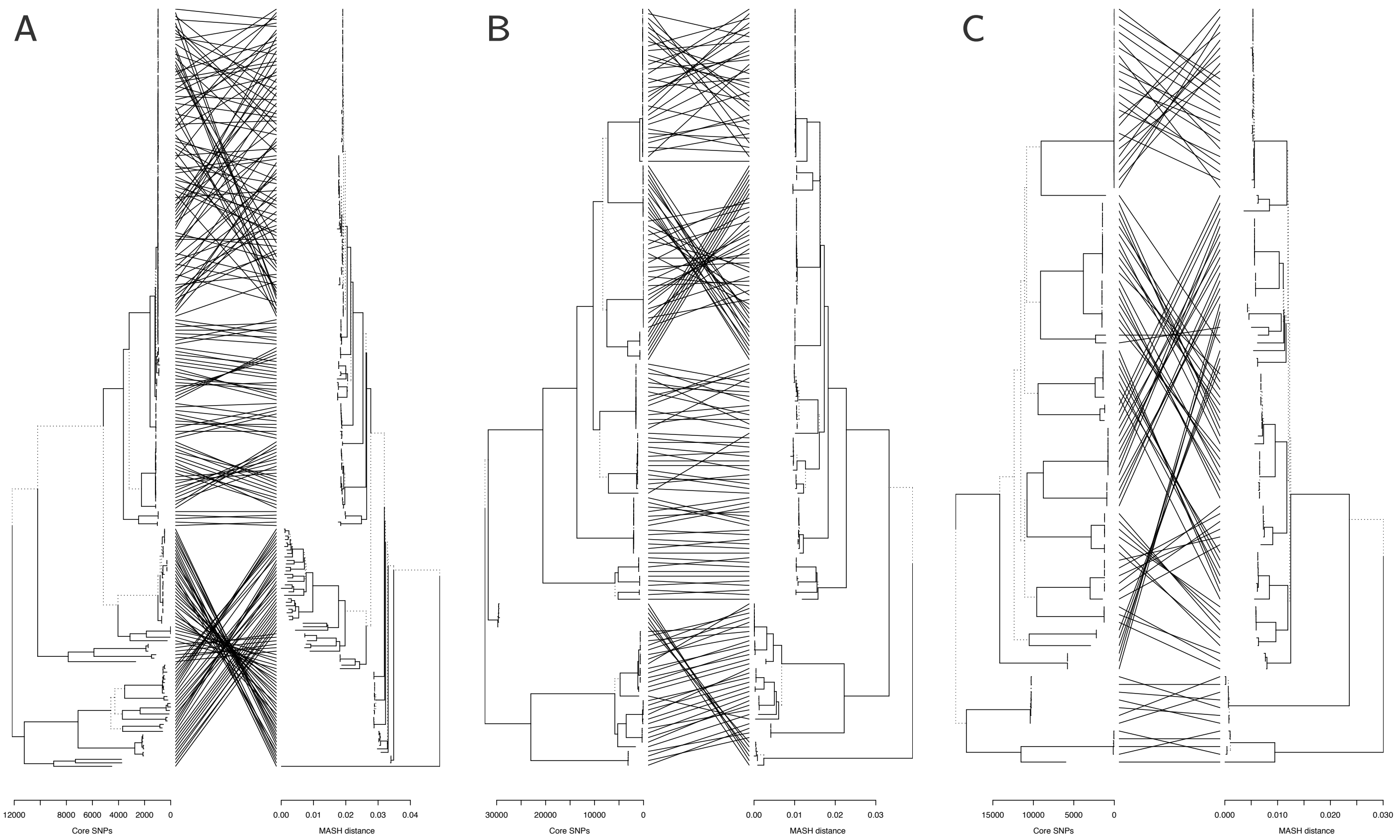

FIGURE S6. Tanglegrams comparing recombination-corrected core phylogenies and read-based whole genome MASH + neighbour joining phylogenies for *a) E. coli*, *b) K. oxytoca* and *c) K. pneumoniae*. Topologically consistent subtrees are rendered with solid branches.

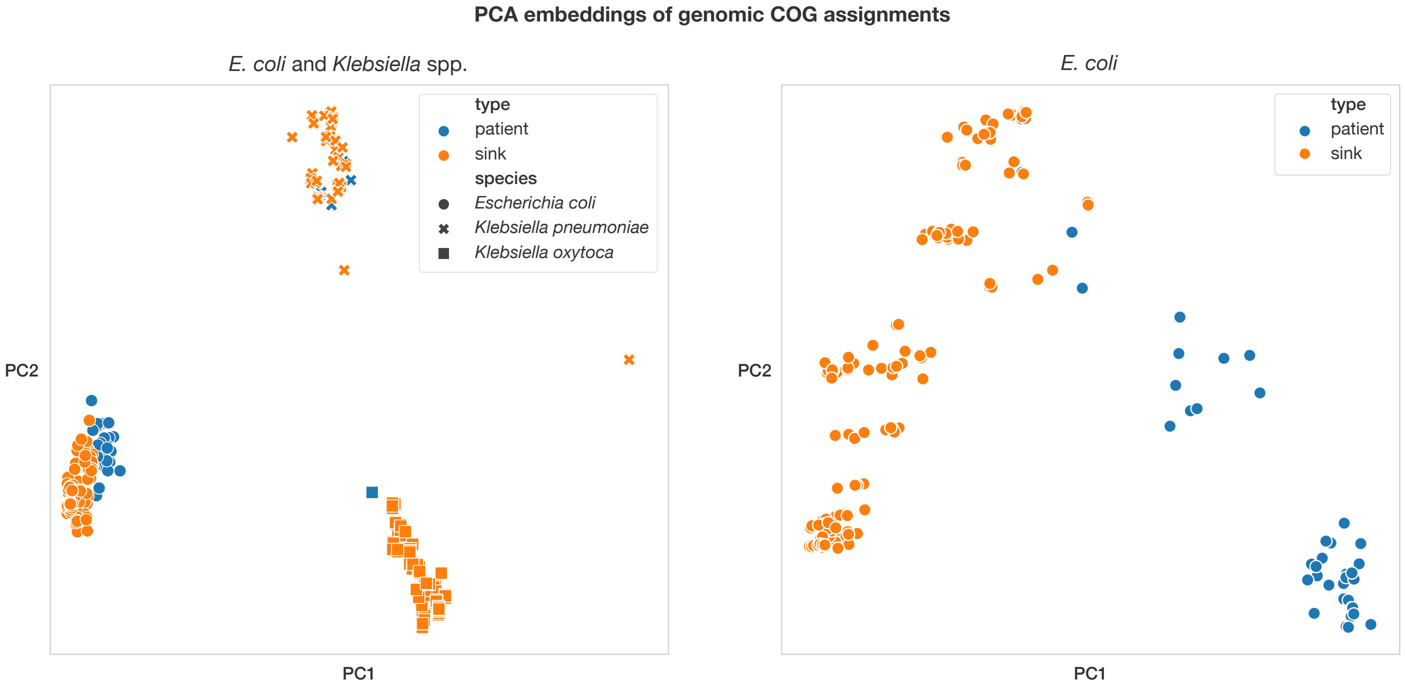

FIGURE S7. Principal component analysis of the functional composition of cultured Enterobacterales isolates using COG annotations from Prokka. Left: *E. coli* and *Klebsiella* spp. Right: *E. coli* only.

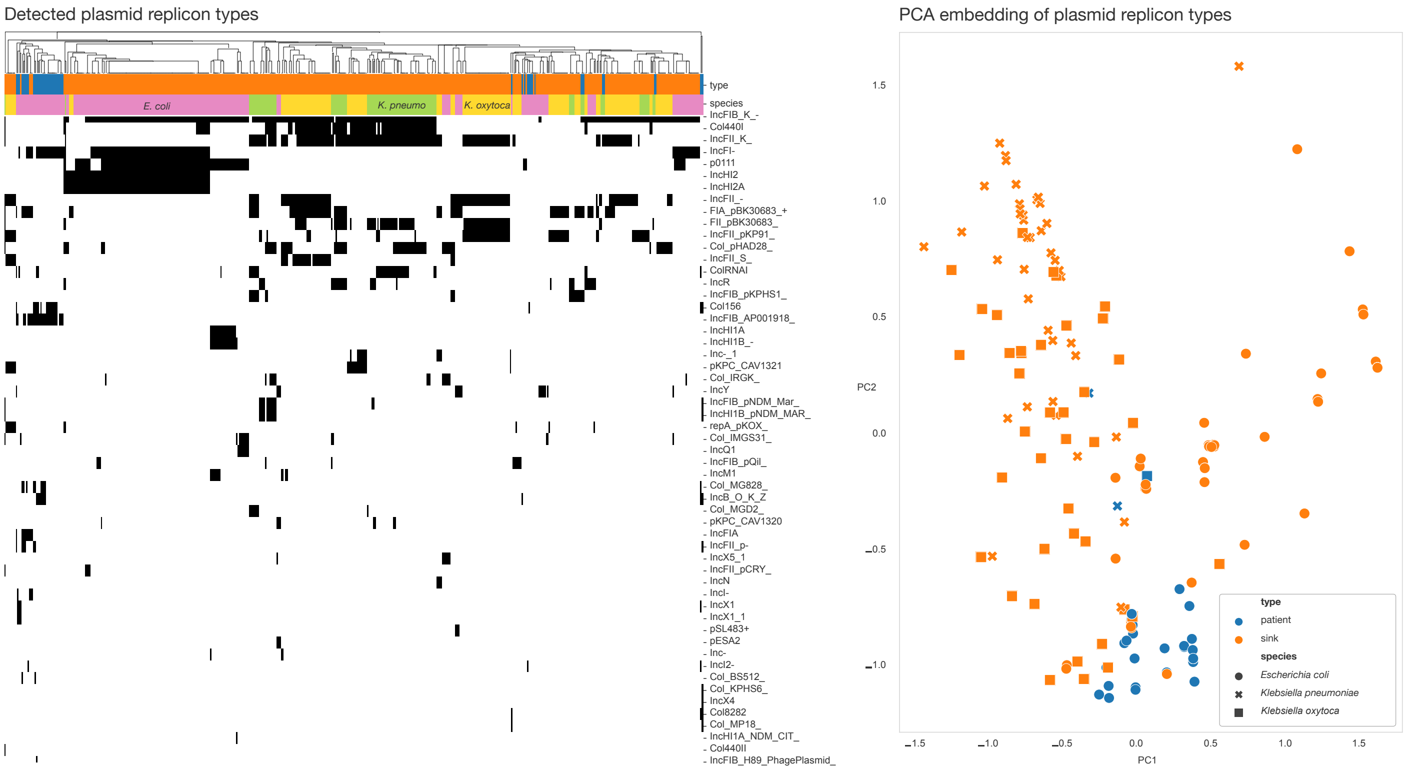

FIGURE S8. Plasmid replicon types detected among sink and clinical isolates. Left: clustered plasmid replicon types as detected by ARIBA using the PlasmidFinder database. Right: principal component analysis of detected replicon types.
